# Supplementary material for: Dynamics of chromosomal target search by a membrane-integrated one-component receptor
Source: PLoS Comput Biol. 2021 Feb 4;17(2):e1008680. doi: 10.1371/journal.pcbi.1008680 (PMC7888679; doi:10.1371/journal.pcbi.1008680)
Supplement: S1 Fig — Fluorescent microscopy images were taken every minute after receptor activation and analyzed for CadC spots for all three E. coli strains. The plot shows the fraction of cells with spots ν(t) as a function of time t after the medium shift to low pH and lysine. (PDF) [file pcbi.1008680.s001.pdf]

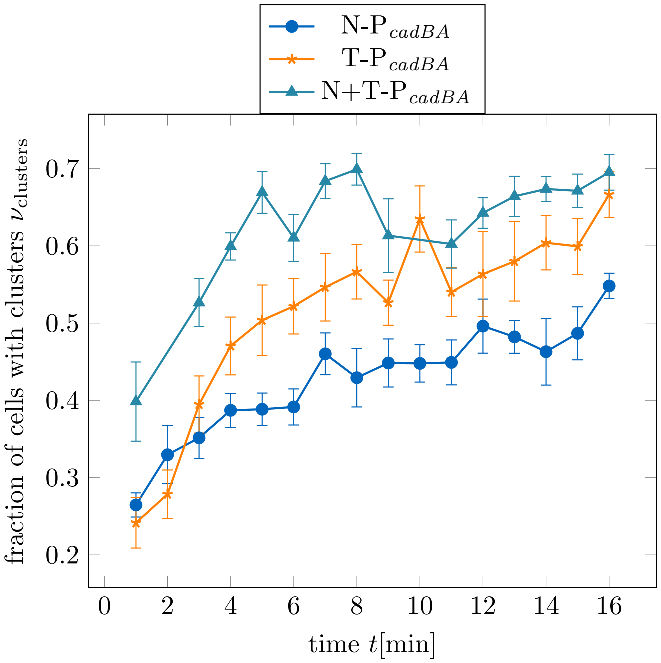

Figure 1: **Dynamics of the target search by CadC.** Fluorescent microscopy images were taken every minute after receptor activation and analyzed for CadC clusters for all three *E. coli* strains. The plot shows the fraction of cells with clusters $\nu(t)$ as a function of time t after the medium shift to low pH and lysine.
