# Supplementary material for: Dynamics of chromosomal target search by a membrane-integrated one-component receptor
Source: PLoS Comput Biol. 2021 Feb 4;17(2):e1008680. doi: 10.1371/journal.pcbi.1008680 (PMC7888679; doi:10.1371/journal.pcbi.1008680)
Supplement: S2 Fig — The MSD of ParB spots was calculated by selecting the closest spots in subsequent image frames and calculating the ensemble-averaged mean square displacement as a function of time lag τ. The dashed lines show the fit to Γτα. For each time lag the mean was taken over 234 to 936 values. (PDF) [file pcbi.1008680.s002.pdf]

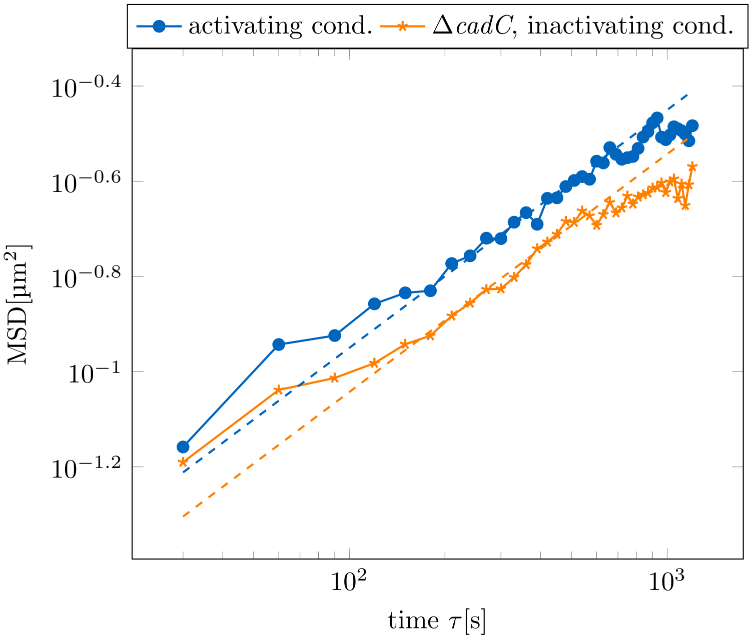

Figure 1: **Mean square displacement of ParB foci.** The MSD of ParB foci was calculated by selecting the closest foci in subsequent image frames and calculating the ensemble-averaged mean square displacement as a function of time lag τ. The dashed lines show the fit to $\mathrm{MSD}(\tau) = \Gamma\tau^{\frac{1}{2}}$. For each time lag the mean was taken over 234 to 936 values.
