# Supplementary material for: Dynamics of chromosomal target search by a membrane-integrated one-component receptor
Source: PLoS Comput Biol. 2021 Feb 4;17(2):e1008680. doi: 10.1371/journal.pcbi.1008680 (PMC7888679; doi:10.1371/journal.pcbi.1008680)
Supplement: S3 Fig — The mean search time τ is plotted against the number of DNA beads NDNA. After an initial increase the search time becomes independent of the polymer length. Simulations were run with the realistic parameter set and varying number of DNA beads. (PDF) [file pcbi.1008680.s003.pdf]

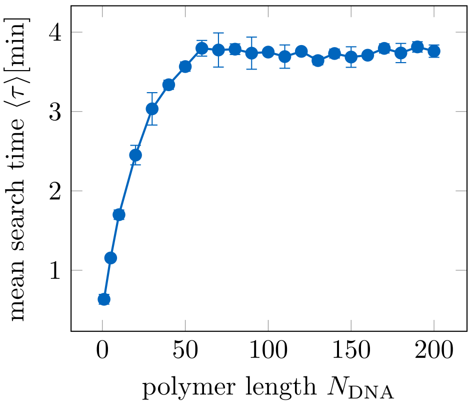

Figure 1: **Polymer length dependence of the search time.** The mean search time τ is plotted against the number of DNA beads N_{DNA}. After an initial increase the search time becomes independent of the polymer length. Simulations were run with the realistic parameter set and varying number of DNA beads.
