# Supplementary material for: Dynamics of chromosomal target search by a membrane-integrated one-component receptor
Source: PLoS Comput Biol. 2021 Feb 4;17(2):e1008680. doi: 10.1371/journal.pcbi.1008680 (PMC7888679; doi:10.1371/journal.pcbi.1008680)
Supplement: S1 Table — Results from fitting the experimentally computed CDF to the sequential reversible model with mixed initial condition (N-PcadBA) and fixed initial condition (T-PcadBA and N+T-PcadBA). The fit parameters α, β and c were used to compute the mean first passage time and the variance with uncertainties obtained from error propagation using the full covariance matrix. (PDF) [file pcbi.1008680.s004.pdf]

Table 1: **Fit results.**

Strain	α[min]	β[min]	c[min^{-1}]	$\langle\tau\rangle$[min]	σ^2[min^2]
N-P$_{cadBA}$	7.87 ± 0.60	0.52 ± 0.13	0.87 ± 0.15	4.84 ± 0.19	49.6 ± 8.8
T-P$_{cadBA}$	4.20 ± 0.26	$6 \times 10^{-15} \pm 0.18$		4.20 ± 0.15	17.6 ± 2.0
N+T-P$_{cadBA}$	2.02 ± 0.16	$1.1 \times 10^{-14} \pm 0.22$		2.02 ± 0.12	4.09 ± 0.66

Results from fitting the experimentally computed CDF to the sequential reversible model with mixed initial condition (N-P$_{cadBA}$) and fixed initial condition (T-P$_{cadBA}$ and N+T-P$_{cadBA}$). The fit parameters α, β and c were used to compute the mean first passage time and the variance with uncertainties obtained from error propagation using the full covariance matrix.
