# Supplementary material for: Dynamics of chromosomal target search by a membrane-integrated one-component receptor
Source: PLoS Comput Biol. 2021 Feb 4;17(2):e1008680. doi: 10.1371/journal.pcbi.1008680 (PMC7888679; doi:10.1371/journal.pcbi.1008680)
Supplement: S2 Table — Covariance matrix of the parameters α, β and c from fitting the experimentally computed CDF to the sequential reversible model with mixed initial condition (N-PcadBA) and fixed initial condition (T-PcadBA and N+T-PcadBA). (PDF) [file pcbi.1008680.s005.pdf]

Table 1: **Covariance matrix.**

Strain	$\sigma_\alpha^2[\mathrm{min}^2]$	$\sigma_\beta^2[\mathrm{min}^2]$	$\sigma_c^2[\mathrm{min}^2]$	$\sigma_{\alpha\beta}[\mathrm{min}^2]$	$\sigma_{\alpha c}[\mathrm{min}^2]$	$\sigma_{\beta c}[\mathrm{min}^2]$
N-P_{cadBA}	0.36	0.016	0.023	0.05	-0.019	-0.05
T-P_{cadBA}	0.059	0.032		-0.033		
N+T-P_{cadBA}	0.026	0.050		-0.030		

Covariance matrix of the parameters α, β and c from fitting the experimentally computed CDF to the sequential reversible model with mixed initial condition (N-P_{cadBA}) and fixed initial condition (T-P_{cadBA} and N+T-P_{cadBA}).
