# Supplementary material for: Dynamics of chromosomal target search by a membrane-integrated one-component receptor
Source: PLoS Comput Biol. 2021 Feb 4;17(2):e1008680. doi: 10.1371/journal.pcbi.1008680 (PMC7888679; doi:10.1371/journal.pcbi.1008680)
Supplement: S1 Text — Derivation of the cumulative distribution function and the first two moments for the sequential reversible process. (PDF) [file pcbi.1008680.s006.pdf]

SUPPLEMENTARY TEXT

Sequential reversible process

We consider a sequential reversible two-step process

$$S_1 \underset{k_2^-}{\overset{k_1^+}{\rightleftharpoons}} S_2 \overset{k_2^+}{\rightarrow} S_3\,.$$

First we consider the general case of N states $n = \{1,\ldots,N\}$. The Master equation for the transition probability $p(n,t|n',t')$ from state n' at time t' to state n at time t reads

$$\frac{\partial}{\partial t} p(n,t|n',t') = k_{n-1}^+ p(n-1,t|n',t') + k_{n+1}^- p(n+1,t|n',t') - (k_n^+ + k_n^-) p(n,t|n',t')\,.$$

Since we are interested in the time τ to reach the final state S_N, we set $k_N^- = 0$, such that S_N becomes an absorbing state. Define $G(m,t)$, the probability that at time t state S_N is not yet reached when starting from state m at time $t = 0$:

$$G(m,t) := \mathbb{P}\{\tau \geq t | n(0) = m\} = \sum_{n=1}^{N} p(n,t|m,0)\,.$$

The distribution of first passage times is obtained from $G(m,t)$, since

$$1 - G(m,t) = \mathbb{P}\{\tau < t | n(0) = m\}\,.$$

$1 - G(1,t)$ is the cumulative distribution function (CDF) of the first passage time, therefore

$$f(\tau) = -\frac{\partial}{\partial t} G(1,t)|_{t=\tau} \tag{1}$$

is the first passage time distribution. We exploit that the transition rates do not depend on time to write

$$G(m,t) = \sum_{n=1}^{N} p(n,0|m,-t)\,.$$

Therefore $G(m,t)$ follows from the evolution of p in the coordinate of the initial state, described by the backward Master Equation

$$\frac{\partial}{\partial t'} p(n,t|n',t') = k_{n'}^+ (p(n,t|n',t') - p(n,t|n'+1,t')) + k_{n'}^- (p(n,t|n',t') - p(n,t|n'-1,t'))\,,$$

which leads to

$$-\frac{\partial}{\partial t} G(m,t) = k_m^+ (G(m,t) - G(m+1,t)) + k_m^- (G(m,t) - G(m-1,t))$$

with initial condition $G(m,0) = 1$ for $m < N$ and $G(N,0) = 0$.

We now go back to the two-step process $N = 3$. Since $G(3,t) = 0$ we have two equations:

$$\begin{aligned}
-\frac{\partial}{\partial t} G(1,t) &= k_1^+ (G(1,t) - G(2,t)) + k_1^- G(1,t) \\
&= k_1^+ (G(1,t) - G(2,t)) \\
-\frac{\partial}{\partial t} G(2,t) &= k_2^+ (G(1,t) - G(3,t)) + k_2^- (G(2,t) - G(1,t)) \\
&= k_2^+ G(1,t) + k_2^- (G(2,t) - G(1,t))
\end{aligned}$$

To solve this initial value problem, we use the Laplace transform

$$\begin{aligned}
F(s) &= \int_0^\infty f(t) e^{-st} \mathrm{d}t \\
f(t) &= \frac{1}{2\pi i} \lim_{T\to\infty} \int_{\gamma - iT}^{\gamma + iT} e^{st} F(s) \mathrm{d}s\,.
\end{aligned}$$

Using $\mathscr{L}(f') = sF(s) - f(0)$ we obtain

$$\begin{aligned}
-sg(1,s) + G(1,0) &= k_1^+(g(1,s) - g(2,s)) \\
-sg(1,s) + 1 &= k_1^+(g(1,s) - g(2,s)) \\
-sg(2,s) + G(2,0) &= k_2^+ g(2,s) + k_2^-(g(2,s) - g(1,s)) \\
-sg(2,s) + 1 &= k_2^+ g(2,s) + k_2^-(g(2,s) - g(1,s)) .
\end{aligned}$$

We solve for $g(1,s)$ and $g(2,s)$ and obtain

$$g(1,s) = \frac{k_2^- + k_1^+ + k_2^+ + s}{k_2^- s + (k_1^+ + s)(k_2^+ + s)}$$

$$g(2,s) = \frac{k_2^- + k_1^+ + s}{k_2^- s + (k_1^+ + s)(k_2^+ + s)}$$

and after back transformation

$$G(1,t) = \frac{e^{-(a+b)t}(-2b + e^{2at}2b + 2a + e^{2at}2a)}{4a} \tag{2}$$

and

$$G(2,t) = \frac{e^{-(a+b)t}(-2(b-k_2^+) + e^{2at}2(b-k_2^+) + 2a + e^{2at}2a)}{4a} \tag{3}$$

with $a = \frac{1}{2}\sqrt{(k_2^- + k_1^+ + k_2^+)^2 - 4k_1^+ k_2^+}$ and $b = \frac{1}{2}(k_2^- + k_1^+ + k_2^+)$. Using eq. (1) we obtain

$$\begin{aligned}
f(\tau) &= \frac{k_1^+ k_2^+}{2a}\left(e^{(a-b)\tau} - e^{-(a+b)\tau}\right) &(4)\\
&= \frac{b^2 - a^2}{2a}\left(e^{(a-b)\tau} - e^{-(a+b)\tau}\right) &(5)
\end{aligned}$$

and for the CDF

$$\mathrm{CDF}(\tau) = 1 - G(1,t) = 1 - \frac{(a-b)e^{-(a+b)\tau} + (a+b)e^{(a-b)\tau}}{2a} .$$

Defining $\alpha = \frac{1}{a+b}$ and $\beta = \frac{1}{b-a}$, the CDF reads

$$\mathrm{CDF}(\tau) = 1 - \frac{\alpha e^{-\frac{\tau}{\alpha}} - \beta e^{-\frac{\tau}{\beta}}}{\alpha - \beta} \tag{6}$$

and the probability density function

$$p(\tau) = \frac{e^{-\frac{\tau}{\alpha}} - e^{-\frac{\tau}{\beta}}}{\alpha - \beta} . \tag{7}$$

When we assume that $k_2^- = 0$, it reduces to

$$\mathrm{CDF}(\tau) = 1 + \frac{k_2^+ e^{-k_1^+\tau} - k_1^+ e^{-k_2^+\tau}}{k_1^+ - k_2^+} . \tag{8}$$

When we assume that k_2^+ is rate limiting, it reduces to

$$\mathrm{CDF}(\tau) = 1 - e^{-k_2^+\tau} . \tag{9}$$

Mixed initial condition

The above first passage time distribution is based on a system that starts initially in state S_1. When we allow a mixed initial state, with a fraction x in state S_2 and a fraction $1-x$ in state S_1, we obtain

$$\begin{aligned}\mathrm{CDF}(\tau) &= \mathbb{P}\{\tau < t | n(0) = xs_2 + (1-x)s_1\} \\ &= x\mathbb{P}\{\tau < t | n(0) = s_2\} + (1-x)\mathbb{P}\{\tau < t | n(0) = s_1\} \\ &= 1 - xG(2,t) - (1-x)G(1,t)\end{aligned}$$

and

$$f(\tau) = -\frac{\partial}{\partial t}(xG(2,t) + (1-x)G(1,t))|_{t=\tau}\,.$$

With $G(1,t)$ and $G(2,t)$ given in eqs. (2) and (3) this yields

$$\mathrm{CDF}(\tau) = 1 - \frac{\alpha e^{-\frac{\tau}{\alpha}} - \beta e^{-\frac{\tau}{\beta}} - \alpha\beta c\left(e^{-\frac{\tau}{\alpha}} - e^{-\frac{\tau}{\beta}}\right)}{\alpha - \beta} \tag{10}$$

and

$$f(\tau) = \frac{e^{-\frac{\tau}{\alpha}} - e^{-\frac{\tau}{\beta}} + c\left(\alpha e^{-\frac{\tau}{\beta}} - \beta e^{-\frac{\tau}{\alpha}}\right)}{\alpha - \beta} \tag{11}$$

with $c = xk_2^+$ and

$$\begin{aligned}\alpha &= 2\left(k_1^+ + k_2^+ + k_2^- - \sqrt{(k_1^+ + k_2^+ + k_2^-)^2 - 4k_1^+k_2^+}\right)^{-1}, \\ \beta &= 2\left(k_2^- + k_1^+ + k_2^+ + \sqrt{(k_2^- + k_1^+ + k_2^+)^2 - 4k_1^+k_2^+}\right)^{-1}.\end{aligned}$$

Moments

For the sequential reversible process with fixed initial condition we use the moments of the probability density function eq. (7) to compute the mean first passage time and the variance as functions of α and β. Since α and β are not independent, we propagate the uncertainty with the whole covariance matrix $\sigma_{ij} = \mathrm{cov}[X_i, X_j] = \mathbb{E}[(X_i - \mathbb{E}[X_i])(X_j - \mathbb{E}[X_j]))]$, such that the uncertainty of a function $y(\alpha, \beta)$ reads

$$\begin{aligned}\Delta y &= \sqrt{\sum_{i=1}^{m}\left(\frac{\partial y}{\partial \sigma_{ii}}\right)^2 + 2\sum_{i=1}^{m-1}\sum_{j=i+1}^{m}\frac{\partial y}{\partial \sigma_{ii}}\frac{\partial y}{\partial x_j}\sigma_{ij}} \\ &= \sqrt{\left(\frac{\partial y}{\partial \alpha}\sigma_\alpha\right)^2 + \left(\frac{\partial u}{\partial \beta}\sigma_\beta\right)^2 + 2\frac{\partial y}{\partial \alpha}\frac{\partial y}{\partial \beta}\sigma_{\alpha\beta}}\,.\end{aligned}$$

We compute the mean (first moment $\mu = \mathbb{E}[X]$)

$$\begin{aligned}\mu &= \alpha + \beta \\ \Delta\mu &= \sqrt{\sigma_\alpha^2 + \sigma_\beta^2 + \sigma_{\alpha\beta}}\end{aligned}$$

and the variance (second central moment $\sigma^2 = \mathbb{E}[(X-\mu)^2]$)

$$\begin{aligned}\sigma^2 &= \alpha^2 + \beta^2 \\ \Delta\sigma^2 &= 2\sqrt{\alpha^2\sigma_\alpha^2 + \beta^2\sigma_\beta^2 + \alpha\beta\sigma_{\alpha\beta}}\,.\end{aligned}$$

For the sequential reversible model with mixed initial condition, we proceed as above using the probability density function in eq. (10). The uncertainty of a function $y(\alpha,\beta,c)$ now reads

$$\Delta y = \sqrt{\left(\frac{\partial y}{\partial \alpha}\sigma_\alpha\right)^2 + \left(\frac{\partial u}{\partial \beta}\sigma_\beta\right)^2 + \left(\frac{\partial y}{\partial c}\sigma_c\right)^2 + 2\frac{\partial y}{\partial \alpha}\frac{\partial y}{\partial \beta}\sigma_{\alpha\beta} + 2\frac{\partial y}{\partial \alpha}\frac{\partial y}{\partial c}\sigma_{\alpha c} + 2\frac{\partial y}{\partial \beta}\frac{\partial y}{\partial c}\sigma_{\beta c}}\,.$$

We again compute the mean

$$\begin{aligned}\mu &= \alpha + \beta - \alpha\beta c\\ \Delta\mu &= \sqrt{\sigma_\alpha^2(1-\beta c)^2 + \sigma_\beta^2(1-\alpha c)^2 + \sigma_c^2\alpha^2\beta^2 + 2\sigma_{\alpha\beta}(1-\alpha c)(1-\beta c) - 2\alpha\beta(\sigma_{\alpha c}(1-\beta c) + \sigma_{\beta c}(1-\alpha c}))\end{aligned}$$

and the variance

$$\begin{aligned}\sigma^2 =& \alpha^2 + \beta^2 - \alpha^2\beta^2c^2\\ \Delta\sigma^2 =& \Big\{4\sigma_\alpha^2\alpha^2(\beta^2c^2-1)^2 + 4\sigma_\beta^2\beta^2(\alpha^2c^2-1)^2 + \sigma_c^2(\beta^2 + \alpha^2c(1-\beta^2c^2))^2 + 8\sigma_{\alpha\beta}\alpha\beta(\alpha^2c^2-1)(\beta^2c^2-1)\\ &+ 8\sigma_{\alpha c}\alpha^3\beta^2c(\beta^2c^2-1) + 8\sigma_{\beta c}\alpha^2\beta^3c(\alpha^2c^2-1)\Big\}^{\frac{1}{2}}\,.\end{aligned}$$
