# Supplementary material for: Dynamics of chromosomal target search by a membrane-integrated one-component receptor
Source: PLoS Comput Biol. 2021 Feb 4;17(2):e1008680. doi: 10.1371/journal.pcbi.1008680 (PMC7888679; doi:10.1371/journal.pcbi.1008680)
Supplement: Attachment — Submitted filename: martini2020dynamics_review.pdf [file pcbi.1008680.s008.pdf]

The authors seek to investigate the mechanisms by which membrane bound receptor molecules may bind to DNA targets in bacterial cells. Precisely, they wish to analyze whether or not the "diffusion and capture" method by which membrane bound proteins bind to DNA target sites while remaining on the membrane is plausible.

To do this, they compare time to binding for the E.Coli CadC receptor to a simulated model of the diffusion and capture method. They simulate a DNA polymer moving on a course-grained three-dimensional grid and the receptor protein moves on the two dimensional surface of this grid. The simulated protein binds to nearby DNA and then may either dissociates or "slide". Monte-Carlo simulations are used to compute the CDF of first passage time to binding in this model, which can be compared to experimental observations.

This work is interesting and useful in that it demonstrates a necessary condition for the proposed mechanism - that the time scales involved are plausible. The work with the biophysical lattice model makes this argument well, especially figure 5.

My main comment with on the paper is that I don't know what purpose was served by the inclusion of the reversible sequential process (equation 2). The authors mention that they make use of a CDF for first-passage time from this model, but in equation 8 make clear that this CDF is coming directly from observation. Ultimately, I could not determine what the inclusion of this model added to the paper's argument.

As I interpret this simple network, the parameters k_1^+ and k_2^+ are determined largely by the diffusive process of the DNA and receptor protein. If this relationship could be explored, then perhaps this simple model could be used to determine likely time-scales based on prior knowledge of the diffusive rates of these species. This would then provide a second method with which to compare observation and model.

My second comment is on the choice of using random movement on a grid in place of diffusion. This is reasonable choice considering computational complexity, but I would have liked to see some analysis of the system as the number of grid-points (N) grows, and perhaps comment on the diffusion limit. This would help show that the conclusions are sound and not an artifact of the simplifying assumption.
