# Supplementary material for: Dynamics of chromosomal target search by a membrane-integrated one-component receptor
Source: PLoS Comput Biol. 2021 Feb 4;17(2):e1008680. doi: 10.1371/journal.pcbi.1008680 (PMC7888679; doi:10.1371/journal.pcbi.1008680)
Supplement: Attachment — Submitted filename: Response to the Reviewers.pdf [file pcbi.1008680.s009.pdf]

Response to the Reviewers' comments

We thank the Reviewers for their time and effort in evaluating our manuscript, and for their helpful comments. In the following, we quote the Reviewers' comments (in blue italic font) and supply a point-by-point response.

Peer Reviewer #1 Evaluations:

This is an intriguing and interesting contribution. This paper describes the that how the trafficking patterns of a membrane bound protein could be critical in establishing the functional contacts with specific cytoplasmic partners. These specific interactions could be key in regulating the cellular response to variety of stimuli as some examples are discussed by authors. Authors focus their study on bacterial membrane and a specific protein residing in the bacterial cell membrane while its binding partner in cytoplasm and through experimental and modeling approach it is shown that accounting for the diffusion and capture steps could possibly be enough to establish functional binding between membrane residing and cytoplasmic residing species. The key novelty here is that Authors consider only a one component system for membrane residing protein i.e., the protein residing in membrane can perform both sensing and response regulation. Investigating this question is certainly interesting and merits publication. Authors also show elegant matching of experimental and computational results. It is also very interesting that even through a simple model authors are able to capture and match the experimental observations.

We thank the Reviewer for the appreciation of our work. We have carefully addressed all questions and comments, as detailed in our point-by-point response below.

However, following points should be addressed:

1. Protein trafficking inside the membrane and around the membrane could be quite complex and may involve a multitude of steps/processes, so Authors should specifically state that their reported time-scale and results are for this specific system. It should also be discussed that what is the life-time of this specific protein in the membrane and in bacterial membranes how other different proteins might behave and what could be time scales or diffusion rates of their movements.

We agree with the Reviewer that these aspects deserve more discussion in the manuscript. Regarding the diffusion of membrane proteins in general, it has been reported that the associated coefficient for two-dimensional diffusion within the cell membrane depends primarily on the size of the transmembrane domain (Kumar et al, Biophys J, 2010; Oswald et al, Biophys J, 2016). A CadC dimer has two transmembrane helices like the membrane protein WALP-KcsA, for which the diffusion constant was measured in multiple studies, yielding values between 0.21 $\mu m^2/s$ (Oswald et al, Biophys J, 2016) and 0.24 $\mu m^2/s$ (Oswald et al, Phys Chem Chem Phys, 2014). In a recent study involving some of us, the relevant CadC diffusion coefficient was also measured, albeit

with a different fluorophor (mNG) than in the present study (Brameyer et al, Commun Biol, 2019). Those measurements yielded values ranging from 0.07 to 0.19 $\mu m^2/s$. For our estimate of the initial model parameter values in the present study, we used the rounded value of 0.2 $\mu m^2/s$, which lies in the range of the quoted values. We then also analyzed how the search time depends on this diffusion coefficient, to determine the sensitivity on this parameter and to predict the behavior for other membrane-integrated proteins. This analysis is shown in Fig. 4D (former panel E). We found that within the typical range of diffusion coefficients of membrane-integrated proteins (Kumar et al, Biophys J, 2010; Oswald et al, Biophys J, 2016), the mean search time varies inversely proportional with the diffusion coefficient.

Regarding the second aspect, the life-time of most membrane proteins and CadC in particular is likely much longer than the timescale of the search process. The role of active degradation should be negligible (relative to dilution by growth), given that the majority of proteins in *E. coli* are not subject to rapid degradation (Erickson et al, Nature, 2017), and individual membrane proteins that have been studied in detail displayed life-times as long as 6 days (Chai et al, Anal Bioanal Chem, 2016). Spontaneous separation of CadC from the membrane or proteolytic cleavage also do not appear to play a role, since CadC is deactivated by external cadaverine (Fritz et al, J Mol Biol, 2009).

In the revised manuscript, we extended the discussion of these aspects, as suggested by the Reviewer.

2. In certain cases, cytosolic diffusion or movements in close proximity to membranes is altered due to either crowding or some other effects could that influence your results?

Since we obtained our model parameters for the DNA and protein dynamics from *in vivo* experiments, we expect that such effects are accounted for by the apparent parameter values (which would be different for *in vitro* experiments where such effects are absent). For clarity, we have added this to our description of the choice of parameters in the revised manuscript.

3. Authors assume that membrane is a uniform medium but, in many cases, membranes have different density regions which could influence the diffusion in those regions. Does changes in membrane density or ease of diffusion will influence your results? Could regions like lipid rafts could affect the results reported here?

Our experiments do not have the spatial and temporal resolution that would be required to detect effects due to heterogeneity of the membrane composition. Accordingly, our model also describes the CadC dynamics only on a coarse-grained level, with an effective diffusion coefficient that corresponds to a mean value over the membrane surface. Our estimate for this mean diffusion coefficient (0.2 $\mu m^2/s$) was obtained from previous *in vivo* experiments in *E. coli*, as described above. How the search time depends on this diffusion coefficient is shown in Fig. 4D of the revised manuscript. We made improvements in the text to make our rationale clearer (see manuscript with marked changes).

4. Authors assume Cadc is in dimer form in the membrane, do you know what is the time scale of this dimerization process? Is it fast or a slow process? and what is the probability of dimerization?

We thank the Reviewer for raising this important question. If CadC dimerization were a slow process, we would indeed have to account for its timescale in the total experimental response time, since dimerization is triggered by the shift to acidic conditions that is our experimental “start signal” for the search process (Buchner et al, J Mol Biol, 2015). However, the following estimate suggests that CadC dimerization is fast enough to not contribute significantly to the total observed response time:

We can estimate the rate of dimerization for two CadC monomers based on the approximate formula *t=R^2/D*(2ln[R/r]+2ln[2]-1)* for the mean time for a particle moving with diffusion constant D on the surface of a sphere of radius R to first encounter a trap of radius r (Singer et al, J Stat Phys, 2006). To estimate the time of dimerization for two moving CadC monomers with diffusion constant D_1 and radius r_1, we insert the relative diffusion constant $D = 2D_1$ and the contact radius $r = 2r_1$. Using a sphere of the same typical surface area as a cell, 4.4μm^2 (Prats et al, 1989, J Bacteriol), we obtain $R = 0.59$ μm. For the radius of a CadC monomer we use $r_1 = 2.4$ nm (Erickson, Buil Proced Online, 2009) and for the CadC monomer diffusion constant $D_1 = 0.34$ μm^2/s, which is a typical value for a membrane protein with a single transmembrane domain (Baranova et al, Nat Microbiol, 2020). We then obtain a mean first encounter time of 5.1s, much faster than the total observed response time of several minutes.

In the revised manuscript, we incorporated the above estimate in the “Materials and methods” section and added a brief comment to the beginning of the “Results” section.

5. Authors use coarse-grained cubic lattice model and kmc in order to model the trafficking and interaction events however, there is a lack of rigor in their description of the computational approach, specifically what type of “time advancing”, strategy they used during their simulations, a pseudo code could be help-full. Authors also describe several relevant parameters however, what is the sensitivity of these parameters on their overall results, how sensitive their results are to the choice of initial conditions.

We thank the Reviewer for this comment regarding the description of our kinetic Monte Carlo approach. For ease of implementation, we did not use the time advancing strategy of the standard Gillespie algorithm (Gillespie, 1976, Comput Phys), but instead a variant referred to as a null-event algorithm, as described in Chatterjee and Vlachos (J Comput Aided Mol Des, 2007). We now state this explicitly in the revised manuscript. As mentioned in the main text, all shown simulations were initialized with random configurations of both the polymer and the protein, such that our results do not depend on a specific initial configuration.

Peer Reviewer #2 Evaluations:

The authors seek to investigate the mechanisms by which membrane bound receptor molecules may bind to DNA targets in bacterial cells. Precisely, they wish to analyze whether or not the "diffusion and capture" method by which membrane bound proteins bind to DNA target sites while remaining on the membrane is plausible.
To do this, they compare time to binding for the E.Coli CadC receptor to a simulated model of the diffusion and capture method. They simulate a DNA polymer moving on a course-grained three-dimensional grid and the receptor protein moves on the two dimensional surface of this grid. The simulated protein binds to nearby DNA and then may either dissociates or "slide". Monte-Carlo simulations are used to compute the CDF of first passage time to binding in this model, which can be compared to experimental observations.
This work is interesting and useful in that it demonstrates a necessary condition for the proposed mechanism - that the time scales involved are plausible. The work with the biophysical lattice model makes this argument well, especially figure 5.

We thank the Reviewer for the careful reading of the manuscript and the appreciation of our work.

My main comment with on the paper is that I don't know what purpose was served by the inclusion of the reversible sequential process (equation 2). The authors mention that they make use of a CDF for first-passage time from this model, but in equation 8 make clear that this CDF is coming directly from observation. Ultimately, I could not determine what the inclusion of this model added to the paper's argument.
As I interpret this simple network, the parameters k 1 + and k 2 + are determined largely by the diffusive process of the DNA and receptor protein. If this relationship could be explored, then perhaps this simple model could be used to determine likely time-scales based on prior knowledge of the diffusive rates of these species. This would then provide a second method with which to compare observation and model.

We thank the Reviewer for pushing us to clarify our rationale. Indeed, we obtain an estimate for the CDF directly from the experimental data, as expressed by Eq. 8. However, we make use of the reversible sequential process to supply useful fit functions for the experimental CDF, which are given by Eq. 9 and Eq. 10. To make this clearer, we added a brief statement below Eq. 8. More generally, a reversible two-step process seems like a sensible zeroth-order description for a search process with two mobile components. *A priori*, it is not clear what the intermediate state corresponds to. If it corresponds to the DNA-binding site having reached the membrane but not yet the CadC dimer, the rates k_{1+} and k_{2+} would correspond to the rate of the DNA-binding site to reach the membrane and the rate of CadC to search the membrane for the DNA-binding site, respectively. Based on our results obtained for this manuscript, we are not yet in a position to make such an identification. However, we are currently planning further research, including also different experimental systems, which will hopefully clarify, amongst other things, whether such an identification is possible.

My second comment is on the choice of using random movement on a grid in place of diffusion. This is reasonable choice considering computational complexity, but I would have liked to see some analysis of the system as the number of grid-points (N) grows, and perhaps comment on the diffusion limit. This would help show that the conclusions are sound and not an artifact of the simplifying assumption.

For the long timescales that we are interested in, i.e. the timescale of the search process, which involves many steps on the grid, the discrete description is a good approximation, since the “hopping rates” are chosen to correctly reflect the underlying continuous diffusion process (see, e.g. C. Gardiner, “Stochastic Methods”, Springer). However, in the ensemble of random initial configurations, there is a small fraction of initial states that are already close to the target state. For this fraction of the ensemble, our lattice model overestimates the search time. However, with our lattice sizes, where this fraction is not even a percent of the total number of initial states, this effect is negligible compared to the biological uncertainties in the system. In the revised manuscript, we now briefly comment on this in the “Materials and methods” section.

Peer reviewer 3:

Understanding the machinery which cells use to respond to signals from their environment is one of the fundamental goals of cell biology. While the most studied sensing systems use both sensors on the cell surface and molecules which transmit that signal to the DNA to change gene expression, in this paper the authors study a system in which one molecule does both tasks. This poses an interesting question: if the molecule doesn't leave the membrane, how does it find the right spot on the hairball of DNA inside the cell?

First, the authors measure the time for the sensor, CadC, to bind the DNA in many cells. They then use a three state model to contextualize the measurements and find mean search times. The authors then build a 3D model which incorporates spatiotemporal dynamics of both DNA and CadC. They do experiments to estimate values for the parameters on which this model depends and show that the model predicts search times close to the measured value, a significant achievement. They then show the model also agrees with a well chosen perturbation experiment, another significant achievement. Taken together, these results suggest that DNA and sensor mobility together allow search times on the same order of magnitude of more conventional sensing systems in which the sensor and signal transmitter are separate molecules, an interesting result.

The study is well executed and its results are of significant biological interest. While I am not an expert in bacteria or signal transduction, it is clear that the experimental and modeling efforts are strong individually. Furthermore, because they are tightly integrated, their impact is even greater than the sum of these parts. It is an example of skills and tools coming together from different realms to gain understanding that could not be achieved by either experiment or modeling alone. The authors have done a wonderful job gathering data which addresses the model parameters directly, and making a model which addresses directly what is measurable. Additionally, the paper is well written and very clear in its goals and execution.

We thank the Reviewer for the appreciation of our work and the careful reading of the manuscript. We have addressed the questions and comments in our point-by-point response below.

Addressing the following point is critical for the conclusions of the paper:

I. In simulation, the authors assume there is one CadC dimer on the cell membrane. However, they state that either 1-3 or 3-5 molecules are present on the membrane. A priori, having 2 or more dimers vs. 1 could impact the search time significantly. The authors should address this point.

The Reviewer is correct in pointing out that the number of CadC dimers is a key parameter affecting the search time. In wild-type *E. coli*, the average copy number of CadC monomers was determined to be in the range 1–3 molecules per cell. For our experiments, we used a (low copy) plasmid-encoded CadC-mCherry fusion with a

slightly elevated average monomer copy number determined to be in the range 3–5 (Brameyer *et al,* Commun Biol, 2019). Hence, it would not have been surprising to find very broad experimental distributions of search times, due to cell-to-cell heterogeneity in the number of CadC dimers. However, it turned out that our simulations with a fixed number of one CadC dimer produced search time distributions that were compatible with the experimental ones, both in terms of their mean values and their width/broadness (using our estimated values for the parameters). This observation suggests that cell-to-cell heterogeneity in the number of CadC dimers does not significantly broaden the search time distribution.

In the revised manuscript, we addressed this point by incorporating the above consideration.

The following suggestions would help improve the clarity of the paper:

We thank the Reviewer for these helpful suggestions.

1. ori should be defined

We changed the first occurrence of “*ori*” into “the origin of replication (*ori*)”.

2. The layout of figure 4 distracts from its main points, and could be improved by some small changes

a. All data should be represented with errorbars to contextualize wiggles (Does NDNA=125 really have a longer search time than the surrounding values?)

As suggested by the Reviewer, we added error bars to all plots in Fig. 4. We also added more simulation runs to reduce the wiggles in Fig. 4C.

b. k1D is included in 4B and its legend, but elsewhere the text says sliding was not included in the simulation. Suggest removing is, as well as associated text in “Biophysical model of the target search process”

We removed k1D from Fig. 4B and from the 'Materials and methods' section, as suggested.

c. 4C is a figure which supports the choice of a parameter value, but does not yield understanding about the biology the authors are addressing. Suggest moving it to the supplement.

We moved Fig. 4C to the supplement.

d. The prominence of the dashed line in 4D detracts from the plotted data. Suggest stopping the y axis at 10^{-1}. An annotation including the value for the relevant experiment would better help the reader contextualize this nice result.

We changed the y-axis as suggested and added red crosses to indicate the experimental value in Fig. 4D.

e. Adding an annotation with the experimental mean time to 4E and 4F would help readers understand that the model closely matches the experiment at the measured parameters, a significant achievement which deserves to be highlighted.

We added red crosses to indicate the experimental value in Fig. 4E and 4F.

f. The dashed line should likely not extend across the axis break in 4F

We corrected the blue dashed line in Fig. 4F to end before the axis break.

3. The authors question if DNA or CadC mobility is "more crucial" or "contributes more". This language is not well defined and detracts from otherwise superb writing. Suggest authors phrase this result in the context of either which timescale limits the search, or which rate the search is more sensitive to changes in.

We are grateful that the Reviewer spotted this imprecise language. We improved the wording in both instances.

4. The naming and value of NDNA are inconsistent (in 4C and text NDNA=100, while in table 2 NDNA=10um)

We thank the Reviewer for pointing out this inconsistency and changed the value of NDNA in Table 2 to 100. We also added "DNA length [beads]" to Table 2 for clarity.
