# Supplementary material for: Dynamics of chromosomal target search by a membrane-integrated one-component receptor
Source: PLoS Comput Biol. 2021 Feb 4;17(2):e1008680. doi: 10.1371/journal.pcbi.1008680 (PMC7888679; doi:10.1371/journal.pcbi.1008680)
Supplement: Attachment — Submitted filename: Response to the Reviewers.pdf [file pcbi.1008680.s010.pdf]

Response to Reviewer 2

Reviewer #2:
The authors use Monte Carlo simulation to model a proposed mechanism for a one-component receptor & transcriptional activator in bacteria, using E. Coli CadC as an example system. Comparison with experimental data makes a convincing case that this mechanism exhibits plausible time-scales, an important necessary condition for the mechanism's accuracy. The paper explains the mechanism and model well. Overall, I think this is a very good use of modeling and simulation.

We thank the Reviewer for evaluating our revised manuscript and for the appreciation of our work.

My only comment is that the use of the two-state stochastic system to fit parameters and thereby compute a mean first passage time still seems unnecessary. The fitted parameters seem to be used just to compute the mean, but this can be computed directly from the observed CDF. There are likely other advantages to having a functional form of the distribution that will be used in future work, and the authors could add some comment as to what these are.

We agree that our revised manuscript still did not fully address this point. We did in fact use the 2-step (3-state) stochastic system for several purposes, but did not fully describe this in the manuscript:

- We used the 2-step model to conceptualize the target search process and to facilitate the mathematical abstraction of the experimental response function as a cumulative first passage time distribution (text around Eqs. 2, 6, and 7).
- We derived explicit functional forms for the first passage time distribution that can be used as simple fit functions for our data, as well as for similar experiments in the future (Eqs. 8 and 9).
- Thereby, we illustrated how distributions resembling the experimental shape arise already from simple assumptions about the search process.
- From the quantitative fits to the experimental response functions, we extracted the mean, the variance, and the statistical errors for both of these quantities using error propagation and the full statistical covariance matrix for the parameters (Table 1, Supplementary Text, and Supplementary Tables 1 and 2 within the Supplementary Text). This was only briefly mentioned in the caption of Table 1 of the main text without reference to the Supplement.

In our newly revised manuscript, we made changes to more clearly express the above points (see marked-up version of the manuscript). Moreover, when more experimental response functions of this type become available for different mutant strains or different model organisms, one could use the simple 2-step model to identify systematic trends in the extracted parameters.
