# Supplementary material for: Dynamics of chromosomal target search by a membrane-integrated one-component receptor
Source: PLoS Comput Biol. 2021 Feb 4;17(2):e1008680. doi: 10.1371/journal.pcbi.1008680 (PMC7888679; doi:10.1371/journal.pcbi.1008680)
Supplement: Attachment — Submitted filename: Reviewer NA comments.docx [file pcbi.1008680.s007.docx]

This is an intriguing and interesting contribution. This paper describes the that how the trafficking patterns of a membrane bound protein could be critical in establishing the functional contacts with specific cytoplasmic partners. These specific interactions could be key in regulating the cellular response to variety of stimuli as some examples are discussed by authors. Authors focus their study on bacterial membrane and a specific protein residing in the bacterial cell membrane while its binding partner in cytoplasm and through experimental and modeling approach it is shown that accounting for the diffusion and capture steps could possibly be enough to establish functional binding between membrane residing and cytoplasmic residing species. The key novelty here is that Authors consider only a one component system for membrane residing protein i.e., the protein residing in membrane can perform both sensing and response regulation. Investigating this question is certainly interesting and merits publication. Authors also show elegant matching of experimental and computational results. It is also very interesting that even through a simple model authors are able to capture and match the experimental observations. However, following points should be addressed:

1. Protein trafficking inside the membrane and around the membrane could be quite complex and may involve a multitude of steps/processes, so Authors should specifically state that their reported time-scale and results are for this specific system. It should also be discussed that what is the life-time of this specific protein in the membrane and in bacterial membranes how other different proteins might behave and what could be time scales or diffusion rates of their movements.
2. In certain cases, cytosolic diffusion or movements in close proximity to membranes is altered due to either crowding or some other effects could that influence your results?
3. Authors assume that membrane is a uniform medium but, in many cases, membranes have different density regions which could influence the diffusion in those regions. Does changes in membrane density or ease of diffusion will influence your results? Could regions like lipid rafts could affect the results reported here?
4. Authors assume Cadc is in dimer form in the membrane, do you know what is the time scale of this dimerization process? Is it fast or a slow process? and what is the probability of dimerization?
5. Authors use coarse-grained cubic lattice model and kmc in order to model the trafficking and interaction events however, there is a lack of rigor in their description of the computational approach, specifically what type of “time advancing”, strategy they used during their simulations, a pseudo code could be help-full. Authors also describe several relevant parameters however, what is the sensitivity of these parameters on their overall results, how sensitive their results are to the choice of initial conditions.
